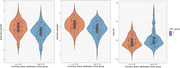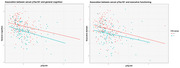# Coronary artery calcium and Alzheimer’s Disease biomarkers in cognitively unimpaired adults

**DOI:** 10.1002/alz.090273

**Published:** 2025-01-09

**Authors:** Sofia Marcolini, Jaime Mondragon, Rozemarijn Vliegenthart, Charlotte Teunissen, Inge M.W. Verberk, Ronald J.H. Borra, Rudi Dierckx, Peter Paul De Deyn

**Affiliations:** ^1^ University Medical Center Groningen, Groningen Netherlands; ^2^ Neurochemistry Laboratory, Department of Clinical Chemistry, Vrije Universiteit Amsterdam, Amsterdam UMC location VUmc, Amsterdam, North Holland Netherlands; ^3^ Neurochemistry Laboratory, Department of Clinical Chemistry, Vrije Universiteit Amsterdam, Amsterdam UMC location VUmc, Amsterdam Netherlands; ^4^ Laboratory of Neurochemistry and Behaviour, University of Antwerp, Antwerp Belgium

## Abstract

**Background:**

Atherosclerosis, the hardening of arterial walls resulting in atherosclerotic plaques, is linked to cognitive dysfunction and an increased risk of cognitive decline. Findings on the impact of high coronary artery calcium (CAC), a subclinical atherosclerosis biomarker, on cognition remain inconsistent. Additionally, its effect on Alzheimer’s Disease (AD) biomarkers has not been previously analysed. This study explores differences in cognitive measures and AD biomarkers in cognitively unimpaired adults with absent or elevated evidence of CAC.

**Method:**

285 cognitively unimpaired adults over fifty were recruited through the Lifelines database, a Dutch population‐based cohort study. Participants underwent non‐enhanced third‐generation dual‐source cardiac computerised tomography to derive total CAC using the Agatston score. They were categorized into two groups: participants with no evidence of CAC (all with a score=0; n=154) and participants with high CAC (with score>300; n=131). Subsequently, participants underwent a neuropsychological assessment and blood was sampled. Domain scores were created for memory, executive functioning, information processing, manual dexterity, and general cognition. Serum AD biomarkers (Aβ40, Aβ42, pTau181, NfL, GFAP) were analysed using a single‐molecule array assay technology. Group differences between the two groups were analysed using ANCOVA models adjusting for main confounders for normally distributed variables, and Mann–Whitney U otherwise.

**Result:**

After excluding participants with MMSE<25, 278 participants (68±9 years, 59% males) remained. CAC scores of participants in the high CAC group had median=667.5 and interquartile range=638.95. These participants, older and with more males, exhibited worse overall cognition [adjusted mean difference=‐0.28 (95% CI=‐0.48,‐0.08)], worse executive functioning [adjusted mean difference= ‐0.21(95% CI=‐0.38,‐0.04)] and higher pTau181 levels (z=‐2.50, p=0.01) compared to the group with absent CAC. No differences were found for all other determined AD biomarkers.

**Conclusion:**

Our results suggest that individuals with high evidence of CAC might be at higher risk of cognitive decline, as evidenced by poorer cognitive function and elevated pTau181 levels. Ongoing investigations aim to validate early cognitive decline imaging markers, including advanced measures of cerebral perfusion and small vessel disease, and explore the relationship between cerebrovascular and AD markers in this population. Additionally, how these relationships vary at different levels of CAC severity is being examined.